# Optimal methods for estimating sports vision in kendo athletes

**DOI:** 10.1038/s41598-020-79534-1

**Published:** 2021-01-11

**Authors:** Daisuke Kudo, Yoshimune Hiratsuka, Mitsuru Nakamura, Yusuke Uchida, Seiji Ono, Akira Murakami

**Affiliations:** 1 Department of Ophthalmology, Juntendo University School of Medicine, Tokyo, Japan; 2 Faculty of Health and Sports Science, Juntendo University, Chiba, Japan; 3 Faculty of Science and Technology, Meijo University, Nagoya, Japan; 4 Faculty of Health and Sport Sciences, University of Tsukuba, Tsukuba, Japan; 5 Department of Ophthalmology, Juntendo University Graduate School of Medicine, Tokyo, Japan

**Keywords:** Physical examination, Quality of life

## Abstract

This study investigated whether the eight standard tests of sports vision used in Japan appropriately reflect sports vision; whether all eight tests are necessary; and if not, which combination yields the optimal model. Participants were kendo practitioners (exercise group, n = 41) and those with no exercise habits (non-exercise group, n = 65). The performance of the two groups on all eight tests were compared. The groups differed in static visual acuity, kinetic visual acuity, and eye/hand coordination. A high correlation (r = 0.75) was observed between static visual acuity and kinetic visual acuity, while contrast sensitivity was moderately correlated with static visual acuity and kinetic visual acuity (r = 0.6), and dynamic visual acuity was moderately correlated with eye/hand coordination (r =  − 0.66). Logistic regression analysis indicated that it is not necessary to conduct all eight tests; the optimal model included static visual acuity, visual reaction time, and eye/hand coordination. Our results suggest that static visual acuity, visual reaction time, and eye/hand coordination are sufficient for assessing visual function in kendo practitioners. For other sports, it may be possible to construct discriminative models using the same method and determine which aspects of visual function and which training methods to emphasise in a given sport.

## Introduction

Different sports require a wide variety of physical functions. Particularly for open skill sports, vision has long been indicated to be crucial. In 1942, Winograd classified baseball players by competitive ability and examined the relationships between batting ability and 11 visual functions^1^. Highly skilled baseball players were found to be significantly superior to baseball players of low skill level in terms of binocular disparity, visual reaction time, and reaction time^1^. Recent studies have also demonstrated that visual function plays a crucial role in sports and that visual function in athletes is superior to that in non-athletes^2–8^. Sport players require a variety of unique visual functions to respond to instantaneous changes in situations that would never occur in daily life. The belief that these visual functions could be improved with training led to the formation of an academic discipline termed sports vision in the United States. In 1978, the American Optometric Association-Sports Vision Section was founded in the United States as the first research organization in the world specializing in sports vision. Various studies have since been conducted on sports vision; these studies have focused on the measurement of vision via special visual function tests and the development, implementation, and assessment of training methods to improve performance on these tests^9–12^. In Japan, a private-sector specialised research institution (the Japan Sports Vision Association) was founded in 1988 to conduct research on sports and vision. Tests of visual functions during sports in Japan have been subjectively selected with reference to the 17 tests proposed by the American Optometric Association-Sports Vision Section based on the visual functions presumed to be necessary for athletic performance. Currently, eight visual function tests are conducted as standard sports vision tests in Japan: static visual acuity (SVA), kinetic visual acuity (KVA), dynamic visual acuity(DVA), contrast sensitivity(CS), ocular motor skill(OMS), depth perception(DP), visual reaction time(VRT), and eye/hand coordination(E/H). These eight tests have formed the basis of most research on the assessment of sports vision in Japan^13–15^.

Many studies have assessed the correlation between measurements on these eight tests and competitive ability among athletes in various sports^13,14^. These studies indicated that visual function is better among athletes than among non-athletes and that highly skilled athletes have better visual function than lower-skilled athletes^5–7^. Other studies have reported that these visual functions could be improved through training^7,9,16–20^.

Although there are many studies that have used these eight tests, no study has evaluated their effectiveness. Moreover, performing these specialised tests requires time and specific devices and equipment, which has prevented their widespread use. Selecting the most optimal option from these eight tests will provide benefits to both athletes and testers. Therefore, we conducted this study with the aims of finding the most suitable test for evaluating sports vision, and determining whether all these tests are necessary and whether some can be omitted from routine testing. Furthermore, we aimed to analyse the data of kendo practitioners to address the above questions. Kendo was selected because although there are many types of sports, kendo is unique in terms of visual function^5,13,14,21,22^. Kendo skills are closely related to not only the ability of dynamic visual acuity but also rapid reaction time, as participants need to acquire appropriate visual information about quick motion from their opponents^13,14,21,22^. Therefore, eight types of visual functions mentioned above would plays an important role in kendo performance. Hence, kendo players were considered suitable to be the study subjects.

## Material and methods

### Participants

The participants comprised 106 healthy individuals (106 men) with no ocular disorders other than ametropia and a bilateral visual acuity of ≥ 1.0 (either unaided or corrected with soft contact lenses during the experiment). Participants were assigned to one of two groups: those who exercised regularly (exercise group) and those who did not (non-exercise group). To eliminate the effects of differences in sports on the results, the study was limited to kendo. Further, to minimise the effects of sex differences on our results, only male participants were recruited. The exercise group comprised 41 individuals (41 men) with a mean age of 35.4 ± 15.7 years (19–65 years) who belonged to a school/university kendo club and who were practicing kendo at least three times per week at the time of the study. The non-exercise group comprised 65 individuals (65 men) with a mean age of 38.1 ± 17.1 years (19–71 years) who had no experience in kendo and were not exercising at all at the time of the study. None of the non-exercise participants played e-sport video games or other kinds of video games. All participants received an explanation of the objective and nature of the study in advance and provided written informed consent to participate. The present study was approved by the Juntendo University Independent Ethics Committee (approval numbers: 30–88) and adhered to the tenets of the Declaration of Helsinki.

### Test methods

The following eight tests were performed by a single examiner under the same conditions for all participants.

#### Static visual acuity (SVA)

Measurement was performed with an AS-4C vision tester (Kowa Company Ltd., Tokyo, Japan), which allows for two modes of measurement: a static visual acuity mode and a kinetic visual acuity mode; the former was used to measure SVA^23–25^. Participants looked into the machine through a binocular eyepiece. The machine was optically configured to display Landolt rings at an effective distance of 30 m, corresponding to a visual acuity value of 1.0. Participants were asked to observe and identify the orientation of the gaps in the displayed Landolt rings; the smallest target size they were able to correctly perceive was used to calculate their visual acuity.

SVA was assessed from 0.1 (low) to 1.6 (high) on a 16-point scale (increments of 0.1). Higher numbers represent better outcomes.

#### Kinetic visual acuity (KVA)

This is the longitudinal (anteroposterior) visual acuity when observing a target moving towards you from an anterior position^23–25^. Measurement was performed with an AS-4C kinetic vision tester (Kowa Company, Ltd., Japan) set to kinetic vision mode, in which optically simulated Landolt rings move towards the participant from a distance of 50 m to a distance of 2 m at a speed of 30 km per hour. The Landolt ring targets were configured such that their apparent size at an effective distance of 30 m from the participant corresponded with a visual acuity of 1.0. Participants pressed a switch when they felt that they were able to correctly perceive the orientation of the gap in the Landolt ring. This would cause the ring to stop moving and the lights in the device to switch off. Participants were then asked to indicate the orientation of the ring they had just perceived, and if their answer was correct, the effective distance at which the ring was stopped was used to calculate the participant’s visual acuity. KVA was assessed from 0.1 (low) to 1.6 (high) on a 16-point scale (increments of 0.1). Two practice trials were conducted, followed by five experimental trials, and the mean value of the five measurements was calculated.

#### Dynamic visual acuity (DVA)

This refers to the horizontal visual acuity when observing a target moving across a screen 90 cm in front of the eyes^24–26^. Measurement was performed with an HI-10 (Kowa Company Ltd.)^24,25^. The edges of the screen formed a 90° viewing angle. The target was a Landolt C moving horizontally in front of the participant; the measured value was the rotational speed (deg/s) of the target when the participant correctly identified on which side the gap was located. The range of rotational speed was 90–270 deg/s. Two practice trials were conducted, followed by five experimental trials, and the mean value of these five measurements was calculated. Higher numbers represent better outcomes.

#### Contrast sensitivity (CS)

Contrast sensitivity is the evaluation of the ability to identify patterns that are not clearly outlined and without distinct contrast. Measurement was conducted with a vision contrast test (Vistech; USA) chart^27^. Panel E (18 cycles/degree) was used to examine how many contrast levels the participants could distinguish from 1 to 8; the score was the number of correct answers (maximum score: 8 points). A chart panel was posted 1 m from the participant. The illumination at the time of measurement was fluorescent lights set to 500 lx. Higher numbers represent better outcomes.

#### Ocular motor skill (OMS)

This indicates the ability to track a fast-moving object with the eyes. Measurement was performed with an ocular motor skill measurement program (Kowa Company, Ltd.; Japan)^25,28^. In this study, rather than directly measuring ocular movement using an eye tracker, we used visual stimuli and button reactions to measure ocular motor skill. During measurement, a 5 mm diameter green dot appeared on a dark green computer screen (dummy target). The dot disappeared after 0.5 s and immediately reappeared in another location; however, on average, one out of every five dots would be yellow (main target). Participants maintained a distance of 30 cm from the computer screen, and were told to follow the dots with their eyes, without moving their head. They were instructed to press a button held in their right hand when a main target appeared. 50 main targets were displayed, and participants were tested on the number and percentage of correct presses (successful presses upon appearance of the main target). Two practice trials were conducted, followed by five experimental trials, and the mean value of these five measurements was calculated. Higher numbers represent better outcomes.

#### Depth perception (DP)

This refers to the sensation of perceiving depth. Measurements were performed with an AS7JS-1 (Kowa Company Ltd.)^24,25,28^. Participants sat at a predefined distance of 2.5 m from the edge of the device used in this measurement. By operating a handheld switch, participants controlled one rod moving forward and backward between two fixed rods at a speed of 25 mm/s. While maintaining the prescribed 2.5 m distance, participants were instructed to press their button when they perceived the centre rod to line up horizontally with the other two. Upon pressing the button, the centre rod stopped moving, and the device recorded the error distance (in mm) between the stopping point and the true centre position. Three practice trials were conducted followed by five experimental trials, and the mean value of the five measurements (cm) was calculated. Lower numbers represent better outcomes.

#### Visual reaction time (VRT)

This refers to the extent of the ability of someone to instantaneously distinguish information. Measurement was performed with specialised software (Kowa Company Ltd.)^24,25,28^. Participants maintained a viewing distance of 30 cm from the computer screen while being measured. A six-digit number was flashed at the centre of the screen for 0.1 s and participants were evaluated on how many of these digits they were able to correctly read. Three numbers were flashed, and participants were scored on how many digits they correctly perceived out of a total of 18 (with 18 points being a perfect score). Two practice trials were conducted followed by five experimental trials, and the mean value of the five measurements (score) was calculated. Higher numbers represent better outcomes.

#### Eye/hand coordination (E/H)

This indicates the ability to determine the position of a flashing light on a panel with the eyes and react quickly with the hand. Measurement was performed using an AcuVision 1000 (International AcuVision Inc., Vancouver, BC, Canada)^25,29^. Participants maintained a viewing distance of 30 cm from the device during measurements. The device comprises 120 red, light-up touch sensors, each 3 cm in diameter, embedded in a 300 × 130 cm rectangular panel. The sensors are configured to turn off if pressed while lit. Participants were instructed to turn off lit sensors by pressing them; if they succeeded in turning off a lit sensor, another would immediately light up in a different location on the board. If the participant was unable to press a lit sensor within 0.9 s, it would automatically turn off, and 0.41 s later, another sensor would light up. Participants repeated this task until they had turned off all 120 lights, which concluded the test. Measurements were carried out after one round of practice. Participants were evaluated on the amount of time (min) it took them to complete the test. The Speed 5 program (1.3 s of lighting time) was used for all participants.

One practice measurement was performed, followed by one real measurement. We assessed the time required to complete the test (min). Lower numbers represent better outcomes. Since the Acuvision1000 is no longer in production and cannot be purchased, the following products can be used as replacements: Supreme Vision (Fujitaka), Dynavision (Dynavision International LLC), the Sports Vision Trainer (Sports Vision Pty Ltd), and the Wayne saccadic fixator (Lafayette Instrument Company).

### Statistical analysis

The data obtained from the above tests were analysed using the following statistical techniques:

Differences in the results of the eight tests between the exercise group and the non-exercise group were examined using Wilcoxon signed-rank test.

Correlations among the results of eight tests were analysed by calculating Pearson correlation coefficients.

We performed multivariable stepwise logistic regression analysis to examine whether all eight of the above tests are necessary to assess sports vision and, if not, which combination of tests would comprise the optimal model. Since we used multivariable logistic analysis, the correlation between the explanatory variables was adjusted. We used a corrected Akaike Information Criterion and the error rate (%) to examine a model using all eight tests and a model selected using the stepwise method. The level of statistical significance was defined as *p* < 0.05.

All statistical analyses were conducted using JMP Statistics Release 11 (SMS, NC).

## Results

### Differences in the results of the eight tests between the exercise group and the non-exercise group

**Figure 1:**
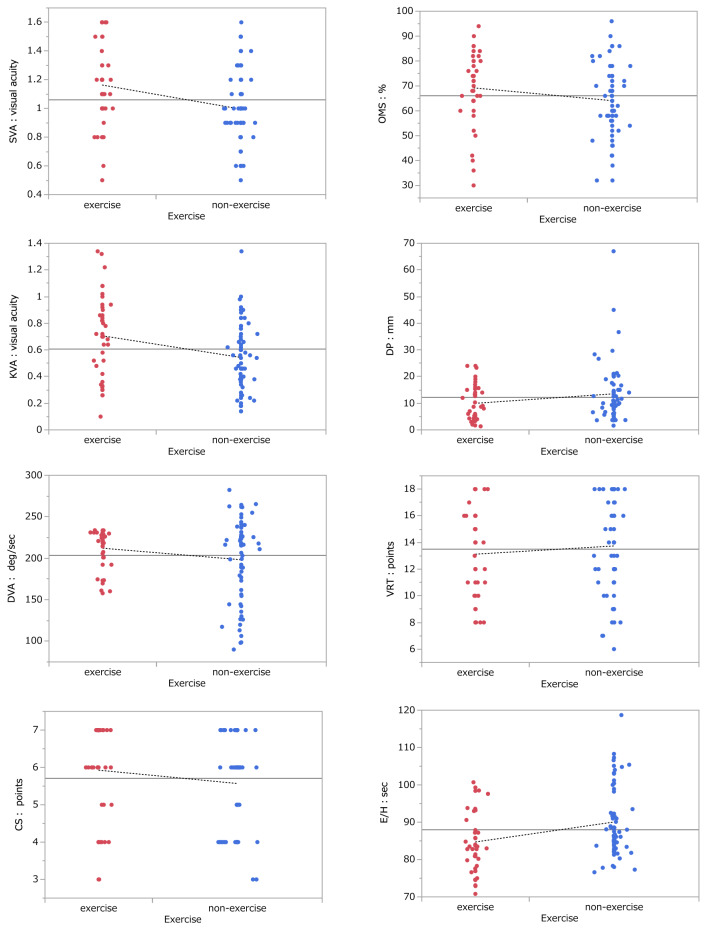
Scatterplots for all 8 tests. Notes: Solid lines indicate the mean of both groups. The mean values for each group are plotted and connected by broken lines. Abbreviations: SVA: static visual acuity, KVA: kinetic visual acuity, DVA: dynamic visual acuity, CS: contrast sensitivity, OMS: ocular motor skill, DP: depth perception, VRT: visual reaction time, E/H: eye hand coordination.

Scatter plots showing the results of the exercise and non-exercise groups on each of the eight tests employed are shown in Fig. 1. The mean values for each group were plotted on the graph and connected by broken lines to make it easier to compare the mean values between the groups. The results of the basic statistics and Wilcoxon tests, including effect sizes, for all eight tests are summarised in Table 1. The exercise group and non-exercise group demonstrated significant differences in three tests: SVA (Wilcoxon test; *p* < 0.01, Effect size; 0.31), KVA (Wilcoxon test; *p* < 0.01, Effect size; 0.27), and E/H (Wilcoxon test; *p* < 0.01, Effect size; 0.28). Of these, SVA, KVA, and E/H allowed rejection of the null hypothesis at the 1% level. These results suggest that SVA, KVA, and E/H are independently highly effective for distinguishing the exercise group and the non-exercise group.

**Table 1:** Basic statistics and Wilcoxon test results for all sports vision tests.

	Overall (n = 106)	Exercise group (n = 41)	Non-exercise group (n = 65)	Effect size	*p* Value
	Mean (SD), Median	Mean (SD), Median	Mean (SD), Median		
SVA	1.06 (0.28), 1	1.16 (0.27), 1.2	1.00 (0.26), 1	0.31	*p* < 0.01
KVA	0.61 (0.29), 0.6	0.71 (0.30), 0.72	0.54 (0.26), 0.54	0.27	*p* < 0.01
DVA	33.93 (6.90), 36.38	35.35 (3.88), 37.38	33.03 (8.16), 36.06	0.12	*p* = 0.20
CS	5.71 (1.25), 6	5.93 (1.31), 6	5.57 (1.20), 6	0.17	*p* = 0.08
OMS	66.06 (14.52), 67	69.22 (14.55), 72	64.06 (14.26), 64	0.21	*p* = 0.03
DP	12.16 (9.40), 10	9.96 (6.65), 8.66	13.54 (10.60), 10.33	0.19	*p* = 0.06
VRT	13.50 (3.48), 14	13.12 (3.35), 14	13.74 (3.56), 14	0.10	*p* = 0.30
E/H	87.99 (9.10), 86.1	84.71 (7.79), 83.5	90.06 (9.21), 87.5	0.28	*p* < 0.01

Abbreviations: SVA: static visual acuity, KVA: kinetic visual acuity, DVA: dynamic visual acuity, CS: contrast sensitivity, OMS: ocular motor skill, DP: depth perception, VRT: visual reaction time, E/H: eye/hand coordination, SD: standard deviation.

### Correlations among the eight tests

We calculated the correlation coefficients among all 26 pairs of the eight tests (Table 2). Of these 26 pairs, the strongest correlation was observed between SVA (r = 0.75) and KVA (r = 0.75). All items were correlated with one another, suggesting that the content measured by these two tests was similar. In addition, CS was moderately correlated with SVA (r = 0.64) and KVA (r = 0.64); the results indicate that the three tests are also relatively similar to one another. A moderate correlation was also observed between DVA (r =  − 0.66) and E/H (r =  − 0.66).

**Table 2:** Correlation coefficients among the eight tests.

	SVA	KVA	DVA	CS	OMS	DP	VRT	E/H
SVA	1.00	0.75	0.42	0.64	0.30	− 0.41	0.30	− 0.45
KVA	0.75	1.00	0.46	0.64	0.27	− 0.31	0.28	− 0.52
DVA	0.42	0.46	1.00	0.42	0.37	− 0.16	0.44	− 0.66
CS	0.64	0.64	0.42	1.00	0.24	− 0.34	0.40	− 0.48
OMS	0.30	0.27	0.37	0.24	1.00	0.06	0.19	− 0.38
DP	− 0.41	− 0.31	− 0.16	− 0.34	0.06	1.00	0.03	0.15
VRT	0.30	0.28	0.44	0.40	0.19	0.03	1.00	− 0.40
E/H	− 0.45	− 0.52	− 0.66	− 0.48	− 0.38	0.15	− 0.40	1.00

Abbreviations: SVA: static visual acuity, KVA: kinetic visual acuity, DVA: dynamic visual acuity, CS: contrast sensitivity, OMS: ocular motor skill, DP: depth perception, VRT: visual reaction time, E/H: eye hand coordination.

### Necessity of the eight tests and the optimal model of test combinations

We compared a logistic model in which all eight tests were used with a logistic model that included only tests selected using the forward stepwise method. This method involves successively adding variables to the model, starting with the variable with the highest chi-square value, and examining the change in the AiCc value after each addition. In the stepwise method, a reference that would minimise the corrected Akaike information criterion was used. With the model using all eight tests, the corrected Akaike information criterion was 146.13, and the error rate was 29.46%. With the model using three tests selected using the stepwise method (SVA, VRT, and E/H), the corrected Akaike information criterion was 135.59, and the error rate was 27.68%; thus, this three-test model was the optimal model. The results of tests of significant differences in all tests in both models are presented in Tables 3 and 4. The variable selection process is presented in Table 5.

**Table 3:** Tests of significant differences in sports vision tests in the eight-test model.

Test value	β	χ^2^	*p* Value
SVA	1.93	2.00	0.16
KVA	0.54	0.18	0.67
DVA	− 0.01	0.07	0.79
CS	− 0.19	0.52	0.47
OMS	0.01	0.46	0.50
DP	− 0.02	0.37	0.54
VRT	− 0.17	4.48	0.03*
E/H	− 0.08	4.47	0.03*

Notes: Model including all eight tests.

Abbreviations: SVA: static visual acuity, KVA: kinetic visual acuity, DVA: dynamic visual acuity, CS: contrast sensitivity, OMS: ocular motor skill, DP: depth perception, VRT: visual reaction time, E/H: eye hand coordination.

**Table 4:** Tests of significant differences in sports vision tests in the three-test model.

Test value	β	χ^2^	*p* Value
SVA	2.16	5.44	0.02*
VRT	− 0.19	6.73	0.01*
E/H	− 0.08	6.47	0.01*

Notes: Model including only tests selected using the stepwise method.

Abbreviations: SVA: static visual acuity, VRT: visual reaction time, E/H: eye hand coordination.

**Table 5:** Variable selection history.

Step	Parameter	Action	Chi-square	*p* Value	p	AICc
1	SVA	Entered	9.768999	0.0018	2	135.813
2	VRT	Entered	3.872309	0.0491	3	134.06
3	E/H	Entered	7.326165	0.0068	4	128.894
4	OMS	Entered	0.309456	0.5780	5	130.789
5	CS	Entered	0.310649	0.5773	6	132.727
6	DP	Entered	0.366175	0.5451	7	134.655
7	KVA	Entered	0.152527	0.6961	8	136.844
8	DVA	Entered	0.06803	0.7942	9	139.167
9	Best	Specific			4	128.894

Abbreviations: SVA: static visual acuity, KVA: kinetic visual acuity, DVA: dynamic visual acuity, CS: contrast sensitivity, OMS: ocular motor skill, DP: depth perception, VRT: visual reaction time, E/H: eye hand coordination.

## Discussion

The results of the present study demonstrated that the eight tests of sports vision are not independent tests, but rather are correlated with one another. In addition, the logistic regression analysis demonstrated two findings: that a model using all eight tests of sports vision cannot be considered optimal for distinguishing between an exercise group and a non-exercise group; and that a model using only SVA, VRT, and E/H is more appropriate.

In Japan, the convention for assessing sports vision has been the eight widely used tests examined in the present study; however, the validity and reliability of this standard method have been called into question. For example, critics have indicated that these eight tests include tests of dynamic vision, for which there is no clear definition or physiological evidence, as well as the fact that the test items, methods, and judgment criteria are not consistent^28^. Others consider it necessary to include only certain measurements, improve measurement methods, and re-examine assessment criteria^28^. These eight tests were originally selected based on the sports vision tests used in the United States, rather than on data or any other definitive proof. The results of the present study demonstrated that the use of all eight of these tests is not necessary to assess sports vision.

We opted to conduct the present study with kendo practitioners. Kendo involves observing the opponent (static visual acuity), wielding a bamboo sword with the hands (eye/hand coordination), and searching for split-second openings in which to strike (visual reaction time). We found that competitive ability in kendo can be assessed by only SVA, VRT, and E/H; this result is consistent with the characteristics of kendo.

In contrast, CS and OMS were suggested to be unnecessary when the participant is a kendo practitioner. These results demonstrate that when considering aptitude for kendo, high SVA, VRT, and E/H performance may represent high visual aptitude. In contrast, it could perhaps be concluded that poor results for CS and OMS do not have much effect on the aptitude for kendo. Thus, the eight tests of sports vision are considered capable of assessing aptitude for a given sport from a visual standpoint.

The results of the present study suggested that applying these methods to sports with different characteristics to those of kendo may identify the optimal combination of tests for a given sport. Focusing the tests to those necessary for a given sport may reduce the time and cost of testing, thereby reducing the burden on the participant.

Focusing on particular tests is also effective for vision training. In the United States, significant effort has been made over the years to improve athletic performance through visual function training under the concept of sports vision^7,19,30^. However, this training was conducted in environments far removed from the conditions of actual competition. Further, while vision is unmistakably an essential element of athletic competition, it has been difficult to assess the extent to which vision training itself independently improves athletic performance. However, based on the findings of the present study, improving SVA, VRT, and E/H through training may improve the performance of kendo practitioners. In other sports as well, tests selected using the method applied in the present study could serve as a guide for evidence-based specific vision training.

The present study had several limitations, which must be considered. First, due to concerns that the characteristics of a given sport would affect the results, the study was limited to kendo. As stated earlier, different sports are likely to require different tests^13,14^; thus, the results of the present study cannot be applied to all sports. However, this study demonstrates a method for evidence-based identification of the visual functions that are specifically important in other sports as well.

Second, we ought to have considered the effects of aging on visual ability. In this study, 44.3% of the participants were over 40 years of age, and it is therefore possible that age-related presbyopia affected our results. A previous study has indicated that visual functions differ according to age^31–33^. Future studies must consider participant performance by age and by the presence or absence of presbyopia.

Third, the effects of differences in the level of kendo proficiency were not accounted for. The participants in the present study varied greatly in their kendo experience, which ranged from several years to several decades. Previous fMRI studies that examined kendo players and non-kendo players showed differences in the brain activity related to vision^34^. Other studies suggesting that experienced and intermediate-level kendo players are characterised by perceptual and behavioural differences^35^ and that gaze position was different between skilled kendo players and beginners^22^. The findings suggest that visual and physical functions evolve by practising kendo and that they may vary according to the skill level. In our study, it was not possible to determine whether the group differences for different visual abilities are due to physical activity or inherent characteristics. In a future study, we would like to investigate further regarding this aspect by adding a new group of “participants who have experience in kendo but are not very good at it” to the experiment to compare players representing different skill levels of skill in kendo.

In conclusion, the present study demonstrates the following: among the eight tests conventionally used to assess standard visual functions in sports, some are unnecessary; further, in kendo, the number of tests can be reduced to three. In the future, the ability to identify which tests are necessary for a given sport may enable more efficient assessment from the perspectives of testing and training.

## Data Availability

The datasets generated and/or analysed during the current study are available from the corresponding author on reasonable request.
